# Glycosphingolipid Changes in Plasma in Parkinson's Disease Independent of Glucosylceramide Levels

**DOI:** 10.1002/mds.29163

**Published:** 2022-07-25

**Authors:** Danielle te Vruchte, Andrea Sturchio, David A. Priestman, Panagiota Tsitsi, Ellen Hertz, Mattias Andréasson, Ioanna Markaki, Kerri‐Lee Wallom, Frances Platt, Per Svenningsson

**Affiliations:** ^1^ Department of Pharmacology University of Oxford Oxford United Kingdom; ^2^ Department of Clinical Neuroscience Neuro Svenningsson, Karolinska Institute Stockholm Sweden; ^3^ James J. and Joan A. Gardner Family Center for Parkinson's Disease and Movement Disorders, Department of Neurology University of Cincinnati Cincinnati Ohio USA; ^4^ Department of Basic and Clinical Neuroscience King's College London London United Kingdom

**Keywords:** Parkinson's disease, glycosphingolipid, pathogenesis

## Abstract

**Background:**

Alteration in glycosphingolipids (GSLs) in Parkinson's disease (PD) still needs to be determined.

**Objectives:**

We evaluated if PD subjects show abnormal GSLs levels compared to healthy controls (HC) and if GSLs correlate with clinical features.

**Methods:**

We analyzed GSLs and glucosylceramide (GlcCer) in plasma using two normal‐phase high‐performance liquid chromatography assays; clinico‐demographic data were extracted.

**Results:**

Eighty PD subjects and 25 HCs were analyzed. Levels of GlcCer, GD1b, Gb4, GalNAcGA1, and b‐series were higher in PD patients than in HCs; total GSLs, GT1b, GM1a, GM3, GM2, and a‐series levels were lower in PD patients than in HCs. Changes in GSLs were present in PD subjects, with GlcCer levels similar to those in HCs. The results were similar after excluding certain *GBA1* mutation carriers. Movement Disorder Society Unified Parkinson's Disease Rating Scale, Part III, correlated with Gb4 and Montreal Cognitive Assessment with GD1b levels.

**Conclusions:**

Multiple GSL abnormalities in plasma were detected in patients with and without GlcCer changes, indicating a broader shift in lipid homeostasis. © 2022 The Authors. *Movement Disorders* published by Wiley Periodicals LLC on behalf of International Parkinson Movement Disorder Society.

Parkinson's disease (PD) is the second most common neurodegenerative disease globally.^1^ Lipid alterations, especially changes in glycosphingolipids (GSLs), have lately gained interest in PD pathogenesis. The role of lipids was highlighted when heterozygote mutations in *GBA1*, encoding the glucosylceramide (GlcCer)‐degrading enzyme glucocerebrosidase (GCase), was identified as the most common genetic risk factor of PD.^2^ GCase catalyzes the catabolism of GlcCer into ceramide and glucose, which in turn can lead to changes in the levels of many downstream metabolites. The mechanism by which *GBA1* mutation carriers increase the risk of PD is not fully understood. Reduced GCase activity has been described in brain tissues of PD patients with and without *GBA1* mutations^3^, ^4^, ^5^ as well as in healthy aging subjects,^3^, ^6^, ^7^ but a recent study showed no correlation between total GCase activity and PD risk.^8^ Overall, the role of GSLs in sporadic PD, independently from *GBA1*‐related disorder, needs broader examination and further clarification.

We, therefore, analyzed the plasma levels of GSLs in PD subjects compared to healthy controls (HC). We identified increased GlcCer in a majority of the PD plasma samples, and therefore, we further stratified the PD population to evaluate if changes in other GSLs were affected by high GlcCer levels, because GlcCer is the precursor of all the GSLs. In addition, as an exploratory outcome, we analyzed whether specific GSLs correlate with clinical features.

## Patients and Methods

### Study Design

Patients' blood samples were retrospectively extracted from a cohort of subjects with parkinsonism (BioPark cohort).^9^ For this study, we included only subjects with a diagnosis of idiopathic PD meeting the United Kingdom Brain Bank criteria.^10^ The study protocol was approved by the local ethical review board, and all participants provided written informed consent. Patients were evaluated while under dopaminergic PD medication. The *GBA1* gene was sequenced by pyrosequencing or screened by TaqMan PCR and confirmed by Sanger sequencing for the following mutations: N370S, T369M, E326K, and L444P.^11^, ^12^ For clinical demographics, see Table 1. The method for GlcCer and GSLs analysis is described in a previous publication,^3^ and the GSLs protocol is available at https://www.protocols.io/view/analysis‐of‐glycosphingolipids‐from‐human‐plasma‐busvnwe6; see Supporting Data for the full nomenclature of GlcCer and GSLs.

**TABLE 1:** General characteristics of the samples and comparison between PD subjects and healthy controls

	PD (N = 80)	HC (N = 25)	*P*‐value
Age (y)	64 (59, 69.75) N = 80	66 (58.50, 68.50) N = 25	ns
Sex (female)	27 (33.75%) N = 80	18 (72.00%) N = 25	0.0011
Disease duration (days)	1062 (488.8, 1823) N = 78	–	–
H&Y	2.0 (2.0, 2.0) N = 80	–	–
MDS‐UPDRS‐III	24.0 (16.0, 29.0) N = 75	–	–
MDS‐UPDRS‐total	47.0 (33.0, 59.0) N = 71	–	–
NMSQ	9.0 (6.0, 13.5) N = 73	–	–
MoCA	26.0 (24.0, 28.0) N = 80	–	–
LEDD (mg)	452.5 (247.5, 629.5) N = 78	–	–
GBA mutation carriers E326K L444P N370S T369M	15 (19.2%) N = 78^a^ 12 – 1 3	5 (26.3%) N = 19 5 – – –	

Data are expressed as median (interquartile range).

^a^ One PD subject was a carrier of both E326K and T369M mutations.

Abbreviations: PD, Parkinson's disease; N, number of subjects; HC, healthy control; ns, not significant; H&Y, Hoehn and Yahr scale; MDS‐UPDRS, Movement Disorder Society Unified Parkinson's Disease Rating Scale; NMSQ, Non‐Motor Symptoms Questionnaire; MoCA, Montreal Cognitive Assessment; LEDD, levodopa equivalent daily dose.

### Aims and Statistical Analysis

With the present study, we aimed to evaluate (1) whether PD subjects demonstrate different GSLs levels compared to HCs and (2) whether GSLs differences, relative to HCs, are present in PD subjects with normal GlcCer levels. We also analyzed, as an exploratory outcome, whether specific GSLs levels correlate with clinical features.

Tests for normal distribution were carried out using Shapiro–Wilk and Kolmogorov–Smirnov tests. Given that most of the data in the PD cohort were nonnormally distributed, a comparison between two groups was performed using Mann‐Whitney *U* test/Fisher's exact test. A comparison between three groups was made using Kruskal–Wallis test and then Dunn's multiple comparison test. Correlations with clinical features were performed with nonparametric partial correlation analysis, adjusted for age, gender, disease duration, and levodopa equivalent daily dose (LEDD); correlations with clinical features stratified by gender were performed with nonparametric partial correlation analysis, adjusted for age, disease duration, and LEDD. For the correlation analysis with clinical features, only subjects with full data were included (LISTWISE exclusion with SPSS). Two‐sided *P*‐value significance was set at 0.05. Mann‐Whitney *U* test/Fisher's exact/Kruskal–Wallis tests for normal distribution tests were carried out using GraphPad Prism (GraphPad Software, version 5.0, Inc., San Diego, CA); nonparametric partial correlation analyses were carried out with SPSS (IBM SPSS Statistics, version 28.0.1.0, IBM Corp, Armonk, NY). Data in tables are expressed in median (interquartile range), frequency, and rho (degree of freedom).

## Results

### Subjects' Characteristics

**FIG 1:**
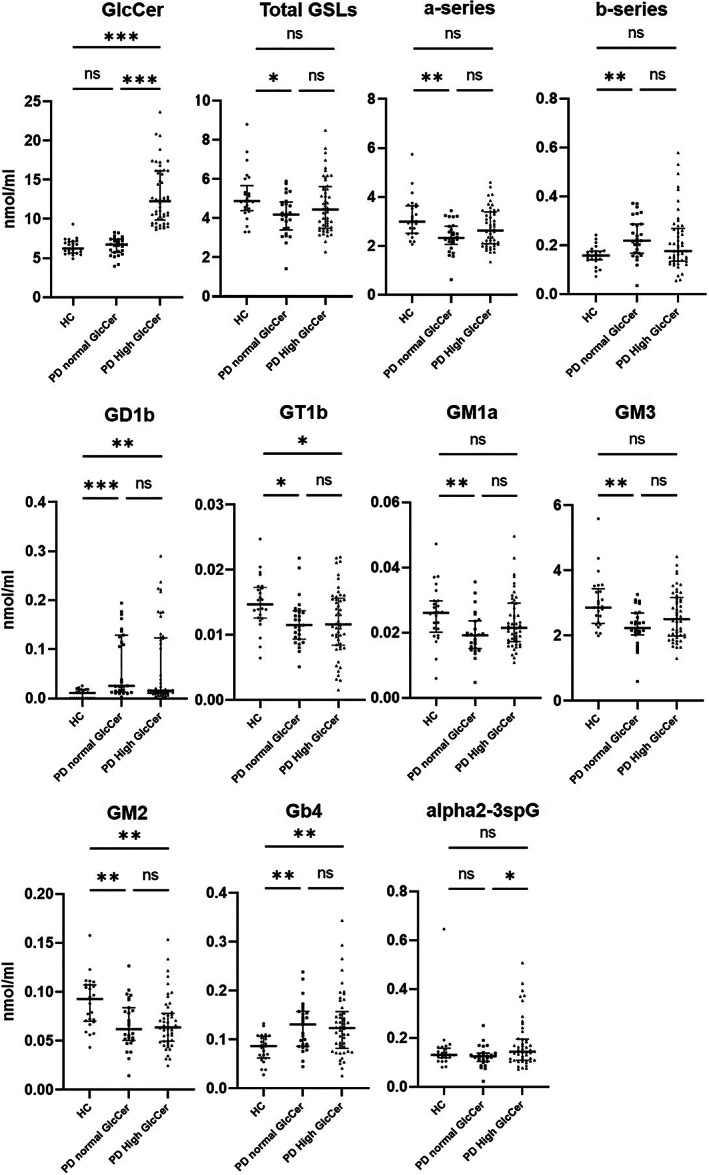
Glycosphingolipid comparison between PD subjects with normal and high glucosylceramide and healthy controls. PD, Parkinson's disease; HC, healthy control; ns, not significant; PD normal, Parkinson's disease with normal glucosylceramide levels; PD high GlcCer, Parkinson's disease with high glucosylceramide levels. *P ≤ 0.05, **P < 0.01, and ***P < 0.001.

A total of 80 PD subjects and 25 HCs were analyzed; clinical and demographic data extracted are presented in Table 1. One outlier value for GM1a and three outlier values for GlcCer were identified in the PD group (Figure 1; Fig. S1) and removed from the analysis; for the comparison between HC vs. PD with normal GlcCer vs. PD with high GlcCer subjects. None of the GlcCer outliers were carriers of the analyzed *GBA1* mutations, whereas the GM1a outlier was a *GBA1* mutation carrier. The GM1a outlier subject was also removed from total GSLs and a‐series values that could have been affected by the GM1a outlier. GA1 levels were not available in the HC group because the data were below the detection limit of the assay. Hoehn and Yahr was significantly higher in female PD cohorts compared to male PD cohorts (mean: 2.2 vs. 1.8, standard deviation ±0.57 vs. 0.44, respectively; *P* = 0.0071); no other differences in clinico‐demographic data have been found.

### Comparison of GSLs Levels between PD Subjects and HCs

GlcCer (*P* < 0.0001), GD1b (*P* < 0.0001), Gb4 (*P* < 0.0001), GalNAcGA1 (*P =* 0.0497), and b‐series (*P =* 0.0070) were higher in PD subjects compared to HCs. On the contrary, total GSLs (*P =* 0.0285), GT1b (*P =* 0.0036), GM1a (*P =* 0.0176), GM3 (*P =* 0.0050), GM2 (*P =* 0.0003), and a‐series (*P =* 0.0042) were lower in PD subjects compared to HCs (Figs. S1 and S2; Table S1). Similar results were obtained after removing known *GBA1* mutation carriers (Table S2). No difference in clinico‐demographic and GSLs data were found between *GBA1* mutation carriers and non‐*GBA1* mutation carriers (Table S3).

GlcCer levels were similar in male and female PD subjects, but both male and female PD cohorts had higher levels compared to controls (both *P* < 0.0001). Other differences in the GSLs stratified by gender versus control are presented in Table S4.

### Comparison of GSLs between HCs versus PD Subjects with Normal GlcCer and PD Subjects with High GlcCer

GlcCer data from HCs were normally distributed (mean: 6.46 nmol/mL, standard deviation ±0.96); therefore, we considered the mean plus 2 standard deviations as a good discriminator for subjects with normal versus high GlcCer levels in the general population. Eleven of 50 (22%) subjects in the high GlcCer group were carriers of *GBA1* mutations (nine E326K, one T369M, and one N370S), whereas 4 of 27 (15%) in the normal GlcCer group were carriers of *GBA1* mutations (two E326K, one T369M, and one E326K + T369M) (*P* = 0.55).

Total GSLs, GM1a, GM3, a‐series, and b‐series were different between HCs and PD subjects with normal GlcCer but not versus PD subjects with high GlcCer; GD1b, GT1b, GM2, and Gb4 were different in both PD subjects with normal and high GlcCer versus HCs (Fig. 1; Table S5). When *GBA1* mutation carriers were removed, there were no changes in GSL levels overall (Table S6).

### Correlation with Clinical Features

Worse score in the Movement Disorder Society Unified Parkinson's Disease Rating Scale (MDS‐UPDRS), Part III, was correlated with higher Gb4 levels (ρ (55): 0.319; *P* = 0.016), whereas better Montreal Cognitive Assessment (MoCA) score was correlated with higher GD1b levels (ρ (55): 0.333; *P =* 0.011) and higher levels of GD1 α (ρ (55): 0.281; *P =* 0.034) (Table S7). After the *GBA1* mutation carriers were removed, only the association with GD1 α was not confirmed. No correlations with other GSLs and GlcCer levels were found. Male PD cohort showed similar results (Table S7); in addition, they showed higher levels of GM1a correlate with MoCA score (ρ (36): 0.341; *P =* 0.034). No correlations with clinical features were found when only the female PD cohort was analyzed.

## Discussion

We have previously reported changes in GSLs levels in postmortem substantia nigra (SN), cerebral spinal fluid (CSF), and serum samples from UK and US biobanks.^3^ In this study we have analyzed plasma samples from a Swedish cohort and replicated parts of our previous findings. Unlike the previous analysis of serum, we also detected increased GlcCer levels in a significant fraction of our subjects. In particular, GSLs changes were more evident in the male PD cohort; however, we cannot exclude that the difference is due to the higher number of male subjects in our cohort rather than a gender difference. Importantly, changes in many of the other GSLs were also present in PD subjects when stratified for GlcCer levels, for instance, even in subjects with GlcCer levels within the range of HCs, suggesting that the presence of GSLs abnormalities was independent of GlcCer accumulation; moreover, certain distinct GSLs species correlate with clinical features of PD.

In our previous study, GlcCer levels were increased in the SN of PD patients,^3^ and two recent studies detected increased GlcCer in the CSF and plasma, respectively, in PD subjects compared to controls.^13^, ^14^ On the contrary, other studies of GlcCer levels in the blood, CSF, and brain have not shown GlcCer accumulation in PD.^3^, ^15^, ^16^, ^17^, ^18^ Although lipid accumulation in PD is still a matter of debate, our results imply that GlcCer accumulation does not represent the only abnormality of GSLs metabolism in PD and may not be the driver of other GSLs changes. Intriguingly, lower expression of the glycosyltransferases B3GALT4 and ST3GAL2 has been found in the SN of PD subjects,^19^ as well as a reduction of the α‐galactosidase, β‐hexosaminidase, β‐galactosidase, and neuraminidase pathways.^3^ In this study, a large fraction, approximately 65% of the PD group, exhibited more than 2 standard deviations higher GlcCer. We also found lower levels of GT1b as previously described in the brain and SN in several studies.^3^, ^20^, ^21^ Contrary to previous analyses, we found higher GD1b levels and lower total GSLs. This difference may be the consequence of different populations; different genetic backgrounds; differences in analytical methods; and sample type, processing, and cohort sample size.

Our analysis showed a positive linear correlation between GD1b and better MoCA score between Gb4 and worse MDS‐UPDRS Part III score when *GBA1* mutation carriers were excluded. These associations were not present in the female PD cohort; however, this cohort had a lower number of subjects, and this could have masked some clinical correlations. GD1b IgM has been found to be associated with dementia,^22^, ^23^ supporting the idea of a possible role of this molecule in cognition. The function of Gb4 is not well known, although a relationship with cell proliferation through the activation of ERK has been described.^24^ Gb4 is not found in the brain, so the relevance of this peripheral GSLs remains unclear. Moreover, Gb4 is highly expressed in the erythrocyte membranes,^25^, ^26^ and hemolytic events can affect the results. Interestingly, GA1 was not found in HCs, because the detected levels were below the detection limit, further supporting the association with neurodegenerative damage.^27^ This study shows that abnormalities in these pathways can be present independently of the accumulation of GlcCer due to genetic mutation or normal aging. Reduction in GCase activity could represent a “second hit” in a landscape of already‐altered GSLs metabolism. However, in our study, most of the patients are carriers of low‐risk mutations,^28^ and even 5 HCs were carriers of low‐risk mutation, namely E326K.

To the best of our knowledge, this is the first study that extensively analyzed specific GSLs in plasma with clinico‐demographic data. Our sample size is larger than that in previous studies, adding strength to our analysis. However, some limitations are present. First, many of the statistically significant differences are minor, with overlap between patients and controls; moreover, male gender was represented more. The finding of GD1b is, in this aspect, encouraging where a subgroup of patients exhibit clearly elevated levels. Second, our subjects have been tested only for selected *GBA1* mutations, and therefore, possibly more *GBA1* mutation carriers are unrecognized in the cohort; however, this eventuality should not affect the results because of the very small numbers of possible mutation carriers of other variants. Moreover, most of the subjects were carriers of low‐risk mutations. Third, we lack data on the levels of GSLs enzymes in this cohort. In addition, the correlation with clinical features does not consider multiple comparisons, so the results should be interpreted with caution. Finally, the cross‐sectional design does not allow the evaluation of the causality of our associations, and replicating these results in another cohort would be important to validate these findings. Nonetheless, this study suggests that the enrollment of smaller, but biochemically well‐characterized, subjects may lead to a greater chance of success when trialing disease‐modifying therapies by reducing heterogeneity.^29^

## Author Roles

1. Research project: A. Conception, B. Organization, C. Execution.
2. Statistical analysis: A. Design, B. Execution, C. Review and critique.
3. Manuscript preparation: A. Writing of the first draft, B. Critical reading and editing.

DtV: 1A, 1B, 1C, 2B, 3B

AS: 1A, 1C, 2A, 2B, 3A

DAP: 1A, 1B, 1C, 2C, 3B

PT: 1C, 2C, 3B

EH: 2C, 3B

MA: 2C, 3B

IM: 1C, 2C, 3B

K‐LW: 1A, 1B, 1C, 2C, 3B

FP: 1A, 1B, 1C, 2C, 3B

PS: 1A, 1B, 1C, 2C, 3A

P.S. had full access to all the data in the study and takes responsibility for the integrity of the data and the accuracy of the data analysis.

## Supporting information


**Table S1.** Glycosphingolipid levels.
**Table S2.** Glycosphingolipid levels excluding *GBA1* mutation carriers.
**Table S3.** Comparison between *GBA1* mutation carriers and noncarriers.
**Table S4.** Glycosphingolipid levels in PD subjects stratified by gender and healthy control.
**Table S5.** Glycosphingolipid levels in PD subjects with normal and high GlcCer.
**Table S6.** Glycosphingolipid levels in PD subjects with normal and high GlcCer excluding *GBA1* mutation carriers.
**Table S7.** Correlations between GSLs and clinical features in PD subjects.
**Figure S1.** Glycosphingolipid comparison between PD subjects and healthy controls. PD, Parkinson's disease; HC, healthy control. *P ≤ 0.05, **P < 0.01, ***P < 0.001, and ****P < 0.0001. Values inside the box are outliers.
**Figure S2.** Glycosphingolipid biosynthetic pathway and representative high‐performance liquid chromatography profile and summary of main findings. HPLC trace of main plasma GSLs not included (GlcCer HPLC profile not shown). Green circles highlight abnormal glycosphingolipids in our analysis; red arrows indicate an increase in PD, whereas blue arrows indicate a decrease in PD.

## Data Availability

The data that support the findings of this study are available from the corresponding authors upon reasonable request.
