# Supplementary material for: Glycosphingolipid Changes in Plasma in Parkinson's Disease Independent of Glucosylceramide Levels
Source: Mov Disord. 2022 Jul 25;37(10):2129–34. doi: 10.1002/mds.29163 (PMC10286748; doi:10.1002/mds.29163)

**GSL nomenclature according to Svennerholm, J. Neurochem. 10, 612-623 (1963).**

GlcCer: Glucosyl ceramide (GlcCer)

LacCer: Lactosyl-Ceramide (Galβ4GlcCer)

Gb3 (Galα4Galβ4GlcCer)

Gb4 (GalNacβ3Galα4Galβ4GlcCer)

GA1 (Galβ3GalNAcβ4Galβ4GlcCer)

GM3 (NeuAcα2-3Galβ4GlcCer)
GD3 (NeuAcα2−8NeuAcα2−3Galβ4GlcCer)
GM2 (GalNacβ4[NeuAcα2−3]Galβ4GlcCer)
GalNAcGA1 (GalNAcβ3Galβ3GalNAcβ4Galβ4GlcCer)

GM1b ([NeuAcα2−3]Galβ3GalNacβ4Galβ4GlcCer)GM1a (Galβ3GalNacβ4[NeuAcα2−3]Galβ4GlcCer)

GD1a (NeuAcα2−3Galβ3GalNacβ4[NeuAcα2−3]Galβ4GlcCer)
GD1b (Galβ3GalNacβ4[NeuAcα2−8NeuAcα2−3]Galβ4GlcCer)
GT1b (NeuAcα2−8NeuAcα2−3Galβ3GalNacβ4[NeuAcα2−3]Galβ4GlcCer) GQ1b(NeuAcα2−8NeuAcα2−3Galβ3GalNacβ4[NeuAcα2−8NeuAcα2−3]Galβ4GlcCer)

GD1alpha (Neu5Acα3Galβ3(Neu5Acα6)GalNAcβ4Galβ4GlcCer)

Paragloboside (pGb) (Galβ4GlcNAcβ3Galβ4GlcCer)

Sialyl(α2-3)paragloboside (NeuAcα2-3Galβ4GlcNAcβ3Galβ4GlcCer)

**Table S1. Glycosphingolipids levels**

|  | **PD (N=80)** | **HC (N=25)** | ***p-*value** |
| --- | --- | --- | --- |
| GlcCer (nmol/ml) | 9.50 (7.30, 12.78) | 6.24 (5.65, 7.13) | <0.0001 |
| Total GSLs (nmol/ml) | 4.28 (3.47, 5.37) | 4.87 (4,38, 5,66) | 0.0285 |
| LacCer (nmol/ml) | 0.43 (0.27, 0.60) | 0.43 (0.36, 0.72) | ns |
| GM1a (nmol/ml) | 0.020 (0.016, 0.026) | 0.026 (0.020, 0.030) | 0.0176 |
| GD1a (nmol/ml) | 0.034 (0.026, 0.044) | 0.032 (±0.023, 0.041) | ns |
| GD1b (nmol/ml) | 0.018 (0.012, 0.12) | 0.011 (±0.0, 0.018) | <0.0001 |
| GT1b (nmol/ml) | 0.011 (0.009, 0.015) | 0.015 (0.013, 0.017) | 0.0036 |
| Gb3 (nmol/ml) | 0.36 (0.26, 0.46) | 0.40 (0.34, 0.48) | ns |
| GM3 (nmol/ml) | 2.39 (2.01, 3.05) | 2.86 (2.38, 3.44) | 0.0050 |
| GM2 (nmol/ml) | 0.06 (0.05, 0.08) | 0.09 (0.07, 0.11) | 0.0003 |
| Gb4 (nmol/ml) | 0.13 (0.09, 0.16) | 0.086 (0.062, 0.11) | <0.0001 |
| pGb (nmol/ml) | 0.18 (0.16, 0.21) | 0.19 (0.16. 0.25) | ns |
| GA1 (nmol/ml) | 0.004 (0.0, 0.005) | **-** | **-** |
| GalNAcGA1 (nmol/ml) | 0.006 (0.003, 0.01) | 0.003 (0.0, 0.011) | 0.0497 |
| GD3 (nmol/ml) | 0.14 (0.11, 0.18) | 0.13 (0.12, 0.15) | ns |
| alpha2-3spG (nmol/ml) | 0.13 (0.11, 0.17) | 0.13 (0.12, 0.16) | ns |
| GM1b (nmol/ml) | 0.058 (0.045, 0.067) | 0.06 (0.054, 0.070) | ns |
| GD1 alpha (nmol/ml) | 0.037 (0.029, 0.042) | 0.04 (0.03, 0,04) | ns |
| o-series (nmol/ml) | 0.55 (0.35, 0.73) | 0.54 (0.467 0.87) | ns |
| a-series (nmol/ml) | 2.50 (2.1, 3.2) | 3.00 (2.52, 3.64) | 0.0042 |
| b-series (nmol/ml) | 0.20 (0.15, 0.28) | 0.16 (0.14, 0.18) | 0.0070 |

PD: Parkinson´s disease; HC: healthy control; GSLs: glycosphingolipids; ns: not significant; N=number of subjects.

**Table S2. Glycosphingolipids levels excluding *GBA1* mutation carriers**

|  | **PD (N=65)** | **HC (N=20)** | ***p-*value** |
| --- | --- | --- | --- |
| GlcCer (nmol/ml) | 9.32 (7.16, 12.52) | 6.64 (5.64, 7.16) | <0.0001 |
| Total GSLs (nmol/ml) | 4.32 (3.5, 5.42) | 4.90 (4,36, 5,84) | ns |
| LacCer (nmol/ml) | 0.45 (0.28, 0.59) | 0.45 (0.36, 0.75) | ns |
| GM1a (nmol/ml) | 0.02 (0.016, 0.025) | 0.026 (0.021, 0.030) | 0.0256 |
| GD1a (nmol/ml) | 0.034 (0.026, 0.044) | 0.034 (0.022, 0.045) | ns |
| GD1b (nmol/ml) | 0.019 (0.011, 0.12) | 0.006 (0.0, 0.018) | 0.0004 |
| GT1b (nmol/ml) | 0.011 (0.009, 0.015) | 0.015 (0.013, 0.017) | 0.0065 |
| Gb3 (nmol/ml) | 0.37 (0.27, 0.46) | 0.40 (0.34, 0.50) | ns |
| GM3 (nmol/ml) | 2.40 (2.02, 3.06) | 2.84 (2.36, 3.48) | 0.0165 |
| GM2 (nmol/ml) | 0.06 (0.05, 0.08) | 0.09 (0.07, 0.11) | 0.0003 |
| Gb4 (nmol/ml) | 0.12 (0.08, 0.16) | 0.086 (0.062, 0.11) | 0.0008 |
| pGb (nmol/ml) | 0.18 (0.15, 0.21) | 0.19 (0.16. 0.25) | ns |
| GA1 (nmol/ml) | 0.004 (0.0, 0.005) | **-** | **-** |
| GalNAcGA1 (nmol/ml) | 0.006 (0.003, 0.01) | 0.003 (0.0, 0.011) | 0.0366 |
| GD3 (nmol/ml) | 0.14 (0.11, 0.18) | 0.14 (0.12, 0.16) | ns |
| alpha2-3spG (nmol/ml) | 0.14 (0.11, 0.17) | 0.13 (0.12, 0.16) | ns |
| GM1b (nmol/ml) | 0.057 (0.045, 0.068) | 0.06 (0.054, 0.07) | ns |
| GD1 alpha (nmol/ml) | 0.037 (0.029, 0.043) | 0.04 (0.03, 0,04) | ns |
| o-series (nmol/ml) | 0.56 (0.36, 0.73) | 0.6 (0.48, 0.89) | ns |
| a-series (nmol/ml) | 2.52 (2.1, 3.2) | 2.95 (2.51, 3.66) | 0.0139 |
| b-series (nmol/ml) | 0.20 (0.15, 0.28) | 0.16 (0.14, 0.18) | 0.0206 |

PD: Parkinson´s disease; HC: healthy control; GSLs: glycosphingolipids ns: not significant; N=number of subjects.

Data expressed as median (interquartile range).

**Table S3. Comparison between *GBA1* mutation carriers and non-carriers.**

|  | ***GBA1* carriers (N=15)** | **Non-carriers (N=65)** | ***p-*value** |
| --- | --- | --- | --- |
| Age (years) | 63 (59, 69) N=15 | 64.0 (58.50, 70.0) N=65 | ns |
| Sex (female) | 8 (53%) N=15 | 19 (29%) N=65 | ns |
| Disease duration (days) | 841 (488, 1371) N=15 | 1242 (489, 1938) N=63 | ns |
| H&Y | 2.0 (2.0, 2.0) N=15 | 2.0 (2.0, 2.0) N=65 | ns |
| MDS-UPDRS-III | 23.50 (11.50, 26.50) N=13 | 24.50 (16.0, 30.75) N=62 | ns |
| MDS-UPDRS-total | 38.50 (26.25, 52.0) N=12 | 49.0 (35.0, 60.0) N=59 | ns |
| NMSQ | 8.0 (6.5, 13.5) N=13 | 9.0 (6.0, 13.75) N=60 | ns |
| MoCA | 26 (23, 28) N=15 | 26 (24, 28) N=65 | ns |
| LEDD (mg) | 300.0 (120.0, 626.0) N=15 | 459.0 (300.0, 640.0) N=63 | ns |
| GlcCer | 10.98 (8.23, 16.18) | 9.32 (7.16, 12.52) | ns |
| Total GSLs (nmol/ml) | 3.98 (3.26, 5.20) | 4.32 (3.50, 5,42) | ns |
| LacCer (nmol/ml) | 0.30 (0.25, 0.63) | 0.45 (0.28, 0.59) | ns |
| GM1a (nmol/ml) | 0.02 (0.017, 0.029) | 0.02 (0.016, 0.026) | ns |
| GD1a (nmol/ml) | 0.029 (0.024, 0.046) | 0.034 (0.026, 0.044) | ns |
| GD1b (nmol/ml) | 0.017 (0.012, 0.13) | 0.019 (0.011, 0.012) | ns |
| GT1b (nmol/ml) | 0.012 (0.008, 0.015) | 0.011 (0.009, 0.015) | ns |
| Gb3 (nmol/ml) | 0.29 (0.25, 0.62) | 0.37 (0.27, 0.46) | ns |
| GM3 (nmol/ml) | 2.36 (1.75, 2.71) | 2.40 (2.02, 3.06) | ns |
| GM2 (nmol/ml) | 0.065 (0.05, 0.09) | 0.063 (0.05, 0.08) | ns |
| Gb4 (nmol/ml) | 0.14 (0.09, 0.16) | 0.12 (0.085, 0.16) | ns |
| pGb (nmol/ml) | 0.19 (0.17, 0.20) | 0.18 (0.15. 0.21) | ns |
| GA1 (nmol/ml) | 0.0 (0.0, 0.005) | 0.004 (0.0, 0.005) | ns |
| GalNAcGA1 (nmol/ml) | 0.004 (0.003, 0.012) | 0.006 (0.003, 0.011) | ns |
| GD3 (nmol/ml) | 0.14 (0.096, 0.17) | 0.14 (0.11, 0.18) | ns |
| alpha2-3spG (nmol/ml) | 0.13 (0.11, 0.19) | 0.14 (0.11, 0.17) | ns |
| GM1b (nmol/ml) | 0.06 (0.05, 0.06) | 0.057 (0.045, 0.068) | ns |
| GD1 alpha (nmol/ml) | 0.034 (0.032, 0.042) | 0.037 (0.029, 0.043) | ns |
| o-series (nmol/ml) | 0.43 (0.35, 0.73) | 0.56 (0.36, 0.73) | ns |
| a-series (nmol/ml) | 2.40 (1.86, 3.02) | 2.52 (2.12, 3.20) | ns |
| b-series (nmol/ml) | 0.18 (0.13, 0.22) | 0.20 (0.15, 0.28) | ns |

PD: Parkinson´s disease; HC: healthy control; GSLs: glycosphingolipids; ns: not significant; N=number of subjects.

Data expressed as median (interquartile range).

**Table S4. Glycosphingolipids levels in PD subjects stratified by gender and healthy control.**

|  | **PD male (N=53)** | **PD female (N=27)** | **HC (N=25)** | ***p-*value** |
| --- | --- | --- | --- | --- |
| Age (years) | 64.0 (57.0, 69.0) N=53 | 66.0 (60.0, 70.0) N=27 | 66.0 (58.5, 68.5) N=25 | ns |
| Disease duration (days) | 1035 (446.0, 1904) N=52 | 1088 (604.3, 1682) N=26 | - | ns^#^ |
| H&Y | 2.0 (1.75, 2.0) N=53 | 2.0 (2.0, 2.0) N=27 | - | 0.0071^#^ |
| MDS-UPDRS-III | 26.0 (17.50, 33.0) N=49 | 19.5 (14.75, 27.0) N=26 | - | ns^#^ |
| MDS-UPDRS-total | 49.0 (35.0, 61.25) N=46 | 39.0 (28.0, 57.0) N=25 | - | ns^#^ |
| NMSQ | 9.0 (6.0, 13.5) N=49 | 9.0 (6.0, 13.75) N=24 | - | ns^#^ |
| MoCA | 26.0 (24.0, 28.50) N=53 | 26.0 (24.0, 28.0) N=27 | - | ns^#^ |
| LEDD (mg) | 452.5 (300.0, 647.5) N=52 | 435.0 (150.0, 577.8) N=26 | - | ns^#^ |
| GlcCer (nmol/ml) | 9.46 (7.08, 14.17) | 10.04 (7.67, 12.67) | 6.24 (5.65, 7.13) | <0.0001^a^ |
| Total GSLs (nmol/ml) | 3.94 (3.40, 4.79) | 5.30 (4.17, 6.25) | 4.87 (4,38, 5,66) | 0.0005^b^ |
| LacCer (nmol/ml) | 0.39 (0.25, 0.56) | 0.53 (0.31, 0.66) | 0.42 (0.36, 0.72) | ns |
| GM1a (nmol/ml) | 0.019 (0.016, 0.023) | 0.024 (0.020, 0.032) | 0.026 (0.020, 0.030) | 0.0010^b^ |
| GD1a (nmol/ml) | 0.032 (0.024, 0.043) | 0.037 (0.029, 0.046) | 0.032 (0.023, 0.041) | ns |
| GD1b (nmol/ml) | 0.022 (0.011, 0.13) | 0.017 (0.013, 0.039) | 0.01 (0.0, 0.018) | 0.0002^a^ |
| GT1b (nmol/ml) | 0.011 (0.008, 0.013) | 0.015 (0.011, 0.018) | 0.015 (0.012, 0.017) | <0.0001^b^ |
| Gb3 (nmol/ml) | 0.33 (0.25, 0.42) | 0.47 (0.35, 0.62) | 0.40 (0.34, 0.48) | 0.0005^c^ |
| GM3 (nmol/ml) | 2.17 (1.91, 2.74) | 3.0 (2.16, 3.42) | 2.86 (2.38, 3.44) | 0.0001^b^ |
| GM2 (nmol/ml) | 0.06 (0.05, 0.07) | 0.08 (0.062, 0.097) | 0.09 (0.07, 0.11) | <0.0001^b^ |
| Gb4 (nmol/ml) | 0.12 (0.08, 0.16) | 0.14 (0.10, 0.17) | 0.086 (0.062, 0.11) | 0.0001^a^ |
| pGb (nmol/ml) | 0.18 (0.16, 0.21) | 0.19 (0.16, 0.20) | 0.19 (0.16. 0.25) | ns |
| GA1 (nmol/ml) | 0.004 (0.0, 0.005) | 0.0009 (0.0, 0.005) | **-** | ns^#^ |
| GalNAcGA1 (nmol/ml) | 0.005 (0.003, 0.0097) | 0.009 (0.004, 0.014) | 0.003 (0.0, 0.011) | 0.0056^d^ |
| GD3 (nmol/ml) | 0.13 (0.11, 0.16) | 0.16 (0.12, 0.19) | 0.13 (0.12, 0.15) | 0.047^e^ |
| alpha2-3spG (nmol/ml) | 0.13 (0.10, 0.15) | 0.16 (0.13, 0.19) | 0.13 (0.12, 0.16) | 0.0374^c^ |
| GM1b (nmol/ml) | 0.06 (0.045, 0.07) | 0.056 (0.045, 0.070) | 0.06 (0.054, 0.094) | ns |
| GD1 alpha (nmol/ml) | 0.036 (0.029, 0.042) | 0.038 (0.032, 0.047) | 0.04 (0.03, 0,04) | ns |
| o-series (nmol/ml) | 0.51 (0.35, 0.69) | 0.65 (0.41, 0.78) | 0.54 (0.46, 0.87) | ns |
| a-series (nmol/ml) | 2.27 (2.01, 2.81) | 3.14 (2.29, 3.60) | 3.00 (2.52, 3.64) | <0.0001^b^ |
| b-series (nmol/ml) | 0.18 (0.14, 0.29) | 0.21 (0.17, 0.24) | 0.16 (0.14, 0.18) | 0.0206^f^ |

PD: Parkinson´s disease; HC: healthy control; GSLs: glycosphingolipids; ns: not significant; N: number of subjects. *p-*value^#^ refers to comparisons were only between male vs. female PD subjects. Data expressed as median (interquartile range).

^a^ Difference was significant between male PD vs. HC and between female PD vs. HC, but not significant between male PD vs. female PD.

^b^ Difference was significant between male PD vs. female PD and between male PD vs. HC, but not significant between female PD vs. HC

^c^ Difference was significant between male PD vs. female PD, but not significant between female PD vs. HC and between male PD vs. HC

^d^ Difference was significant between male PD vs. female PD and between female PD vs. HC, but not significant between male PD vs. HC

^e^ No significant between groups after multiple comparison.

^f^ Difference was significant between female PD vs. HC, but not significant between male PD vs. female PD and between male PD vs. HC.

**Table S5. Glycosphingolipids levels in PD subjects with normal and high GlcCer**

|  | **PD with normal GlcCer (N=27)** | **PD with high GlcCer (N=50)** | **HC**  **(N=25)** | ***p-*value** |
| --- | --- | --- | --- | --- |
| Age (years) | 69.0 (59.0, 71.0) N=27 | 63.5 (54.75, 68.25) N=50 | 66.0 (58.5, 68.5) N=25 | ns |
| Sex (female) | 8 (29.6%) N=27 | 17 (34%) N=50 | 18 (72%) N=25 | <0.05^a^ |
| Disease duration (days) | 1113 (508.0, 2047) N=27 | 1062 (488.5, 1684) N=49 | - | ns^#^ |
| H&Y | 2.0 (2.0, 2.0) N=27 | 2.0 (2.0, 2.0) N=50 | - | ns^#^ |
| MDS-UPDRS-III | 24.0 (16.0, 34.5) N=25 | 24.0 (15.0, 28.0) N=47 | - | ns^#^ |
| MDS-UPDRS-total | 45.0 (33.0, 59.0) N=25 | 49.0 (33.0, 59.0) N=43 | - | ns^#^ |
| NMSQ | 8.5 (5.5, 10.5) N=26 | 9.5 (6.0, 14.0) N=44 | - | ns^#^ |
| MoCA | 26.0 (24.0, 28.0) N=27 | 26.0 (23.0, 28.25) N=50 | - | ns^#^ |
| LEDD (mg) | 400.0 (240.0, 582.5) N=27 | 455.0 (247.5, 639.0) N=49 | - | ns^#^ |
| GlcCer (nmol/ml) | 6.74 (5.75, 7.43) | 12.27 (9.86, 16.16) | 6.24 (5.65, 7.13) | <0.0001^b^ |
| Total GSLs (nmol/ml) | 4.21 (3.47, 4.87) | 4.45 (3.49, 5.61) | 4.87 (4,38, 5,66) | 0.0350^c^ |
| LacCer (nmol/ml) | 0.45 (0.33, 0.59) | 0.41 (0.26, 0.63) | 0.42 (0.36, 0.72) | ns |
| GM1a (nmol/ml) | 0.019 (0.015, 0.024) | 0.021 (0.017, 0.029) | 0.026 (0.020, 0.030) | 0.0106^c^ |
| GD1a (nmol/ml) | 0.033 (0.026, 0.041) | 0.034 (0.026, 0.046) | 0.032 (0.023, 0.041) | ns |
| GD1b (nmol/ml) | 0.025 (0.013, 0.13) | 0.016 (0.011, 0.12) | 0.01 (0.0, 0.018) | 0.0001^a^ |
| GT1b (nmol/ml) | 0.011 (0.009, 0.014) | 0.012 (0.008, 0.016) | 0.015 (0.012, 0.017) | 0.0130^a^ |
| Gb3 (nmol/ml) | 0.40 (0.26, 0.45) | 0.36 (0.26, 0.50) | 0.40 (0.34, 0.48) | ns |
| GM3 (nmol/ml) | 2.24 (2.03, 2.70) | 2.505 (1.99, 3.17) | 2.86 (2.38, 3.44) | 0.0048^c^ |
| GM2 (nmol/ml) | 0.06 (0.05, 0.08) | 0.06 (0.05, 0.08) | 0.09 (0.07, 0.11) | 0.0014^a^ |
| Gb4 (nmol/ml) | 0.13 (0.09, 0.16) | 0.12 (0.08, 0.16) | 0.086 (0.062, 0.11) | 0.0008^a^ |
| pGb (nmol/ml) | 0.17 (0.14, 0.20) | 0.19 (0.17, 0.22) | 0.19 (0.16. 0.25) | ns |
| GA1 (nmol/ml) | 0.004 (0.0, 0.005) | 0.003 (0.0, 0.005) | **-** | ns^#^ |
| GalNAcGA1 (nmol/ml) | 0.004 (0.003, 0.009) | 0.006 (0.004, 0.013) | 0.003 (0.0, 0.011) | ns |
| GD3 (nmol/ml) | 0.15 (0.12, 0.19) | 0.13 (0.11, 0.17) | 0.13 (0.12, 0.15) | ns |
| alpha2-3spG (nmol/ml) | 0.12 (0.10, 0.14) | 0.14 (0.11, 0.19) | 0.13 (0.12, 0.16) | 0.0356^d^ |
| GM1b (nmol/ml) | 0.06 (0.04, 0.07) | 0.058 (0.048, 0.069) | 0.06 (0.054, 0.094) | ns |
| GD1 alpha (nmol/ml) | 0.033 (0.029, 0.041) | 0.037 (0.030, 0.045) | 0.04 (0.03, 0,04) | ns |
| o-series (nmol/ml) | 0.58 (0.41, 0.71) | 0.55 (0.36, 0.75) | 0.54 (0.46, 0.87) | ns |
| a-series (nmol/ml) | 2.36 (2.11, 2.83) | 2.63 (2.1, 3.4) | 3.00 (2.52, 3.64) | 0.0049^c^ |
| b-series (nmol/ml) | 0.22 (0.17, 0.29) | 0.18 (0.14, 0.27) | 0.16 (0.14, 0.18) | 0.0056^c^ |

PD: Parkinson´s disease; HC: healthy control; GSLs: glycosphingolipids; ns: not significant; N=number of subjects. *p-*value^#^ refers to comparison between PD with normal vs. high GlcCer levels. Data expressed as median (interquartile range).

^a^ Difference was significant between PD with high GlcCer vs. HC and between PD with normal GlcCer vs HC, but not significant between PD with normal vs. high GlcCer.

^b^ Difference was significant between PD with high GlcCer vs. HC and between PD with high GlcCer and PD with normal GlcCer, but not significant between PD with normal GlcCer vs. HC.

^c^ Difference only significant between PD with normal GlcCer vs. HC.

^d^ Difference only significant between PD with normal GlcCer vs. PD with high GlcCer.

**Table S6. Glycosphingolipids levels in PD subjects with normal and high GlcCer excluding *GBA1* mutation carriers.**

|  | **PD with normal GlcCer (N=23)** | **PD with high**  **GlcCer (N=39)** | **HC (N=20)** | ***p-*value** |
| --- | --- | --- | --- | --- |
| Age (years) | 69.0 (59.0, 71.0) N=23 | 64.0 (54.0, 68.0) N=39 | 65.0 (55.25, 68.75) N=20 | ns |
| Sex (female) | 6 (26.9%) N=23 | 11 (28.2%) N=39 | 13 (65%) N=20 | <0.05^a^ |
| Disease duration (days) | 1689 (643.0, 2326.0) N=23 | 1224 (474.8, 1769.0) N=38 | - | ns^#^ |
| H&Y | 2.0 (2.0, 2.0) N=23 | 2.0 (2.0, 2.0) N=39 | - | ns^#^ |
| MDS-UPDRS-III | 25.0 (16.0, 35.5) N=22 | 24.0 (16.5, 28.5) N=37 | - | ns^#^ |
| MDS-UPDRS-total | 50.5 (34.0, 61.25) N=22 | 49.0 (34.5, 59.0) N=34 | - | ns^#^ |
| NMSQ | 9.0 (6, 10.5) N=22 | 10.0 (5.0, 14.0) N=35 | - | ns^#^ |
| MoCA | 26.0 (24.0, 28.0) N=23 | 26.0 (23.0, 29.0) N=39 | - | ns^#^ |
| LEDD (mg) | 440.0 (240.0, 640.0) N=23 | 457.0 (296.5, 620.5) N=38 | - | ns^#^ |
| GlcCer (nmol/ml) | 6.64 (5.5, 7.43) | 11.55 (9.43, 15.90) | 6.64 (5.63, 7.16) | <0.0001^b^ |
| Total GSLs (nmol/ml) | 4.16 (3.2, 4.8) | 4.63 (3.66, 5.60) | 4.90 (4,36, 5,84) | 0.0301^c^ |
| LacCer (nmol/ml) | 0.44 (0.28, 0.57) | 0.46 (0.29, 0.65) | 0.45 (0.36, 0.75) | ns |
| GM1a (nmol/ml) | 0.019 (0.015, 0.024) | 0.021 (0.017, 0.028) | 0.026 (0.021, 0.030) | 0.0315^c^ |
| GD1a (nmol/ml) | 0.036 (0.026, 0.041) | 0.034 (0.027, 0.046) | 0.034 (±0.022, 0.044) | ns |
| GD1b (nmol/ml) | 0.025 (0.012, 0.12) | 0.016 (0.010, 0.12) | 0.006 (±0.0, 0.018) | 0.0015^a^ |
| GT1b (nmol/ml) | 0.011 (0.01, 0.014) | 0.011 (0.008, 0.016) | 0.015 (0.012, 0.017) | 0.0230^a^ |
| Gb3 (nmol/ml) | 0.37 (0.26, 0.45) | 0.37 (0.29, 0.50) | 0.39 (0.34, 0.50) | ns |
| GM3 (nmol/ml) | 2.16 (1.77, 2.56) | 2.65 (2.02, 3.14) | 2.84 (2.36, 3.48) | 0.0053^c^ |
| GM2 (nmol/ml) | 0.06 (0.05, 0.08) | 0.06 (0.05, 0.08) | 0.09 (0.07, 0.11) | 0.0012^a^ |
| Gb4 (nmol/ml) | 0.13 (0.09, 0.15) | 0.12 (0.07, 0.16) | 0.08 (0.06, 0.11) | 0.0055^a^ |
| pGb (nmol/ml) | 0.17 (0.13, 0.20) | 0.19 (0.16, 0.22) | 0.19 (0.16. 0.25) | 0.0441^d^ |
| GA1 (nmol/ml) | 0.004 (0.0, 0.005) | 0.003 (0.0, 0.005) | **-** | ns^#^ |
| GalNAcGA1 (nmol/ml) | 0.006 (0.003, 0.009) | 0.006 (0.004, 0.013) | 0.003 (0.0, 0.011) | ns |
| GD3 (nmol/ml) | 0.15 (0.12, 0.18) | 0.13 (0.11, 0.18) | 0.14 (0.12, 0.16) | ns |
| alpha2-3spG (nmol/ml) | 0.12 (0.10, 0.14) | 0.14 (0.11, 0.19) | 0.13 (0.12, 0.16) | ns |
| GM1b (nmol/ml) | 0.06 (0.04, 0.07) | 0.06 (0.05, 0.07) | 0.06 (±0.05, 0.07) | ns |
| GD1 alpha (nmol/ml) | 0.037 (0.029, 0.042) | 0.04 (0.03, 0.049) | 0.039 (0.034, 0,044) | ns |
| o-series (nmol/ml) | 0.54 (0.35, 0.69) | 0.58 (0.38, 0.75) | 0.60 (0.48, 0.89) | ns |
| a-series (nmol/ml) | 2.29 (1.91, 2.67) | 2.78 (2.1, 3.4) | 2.95 (2.51, 3.66) | 0.0061^c^ |
| b-series (nmol/ml) | 0.22 (0.16, 0.29) | 0.19 (0.14, 0.28) | 0.16 (0.14, 0.18) | 0.0367^c^ |

PD: Parkinson´s disease; HC: healthy control; GSLs: glycosphingolipids; ns: not significant; N=number of subjects. *p-*value^#^ refers to comparison between PD with normal vs. high GlcCer levels. Data expressed as median (interquartile range).

^a^ Difference was significant between PD with high GlcCer vs. HC and between PD with normal GlcCer vs HC, but not significant between PD with normal vs. high GlcCer.

^b^Difference was significant between PD with high GlcCer vs. HC and between PD with high GlcCer and PD with normal GlcCer, but not significant between PD with normal GlcCer vs. HC.

^c^ Difference only significant between PD with normal GlcCer vs. HC.

^d^ No significant between groups after multiple comparison.

**Table S7. Correlations between GSLs and clinical features in PD subjects**

|  | **MDS-UPDRS part III** | **MoCA** |
| --- | --- | --- |
| **PD cohort** | Gb4:  Rho (55): 0.319;  *p* =0.016 | GD1b:  Rho (55): 0.333;  *p*: 0.011  GD1 alpha:  Rho (55): 0.281;  *p*: 0.034 |
| **PD cohort without *GBA1* mutation carriers** | Gb4:  Rho (46): 0.389;  *p*: 0.006 | GD1b:  Rho (46): 0.319;  *p*: 0.027 |
| **Male PD cohort** | Gb4:  Rho (36): 0.426;  *p*: 0.008 | GD1b:  Rho (36): 0.334;  *p*: 0.041  GD1 alpha:  Rho (36): 0.355;  *p*: 0.029  GM1a:  Rho (36): 0.341;  *p*: 0.034 |

PD: Parkinson´s disease; HC: healthy control; MDS-UPDRS: Movement Disorder Society Unified Parkinson's Disease Rating Scale; NMSQ: Non-Motor Symptoms Questionnaire; MoCA: Montreal Cognitive Assessment.

All the analyses were adjusted for age, gender, disease duration, and LEDD, except for male PD cohort, that was adjusted for for age, disease duration, and LEDD.

Data are expressed in rho (degree of freedom).

**Figure S1.**

**Figure S2**


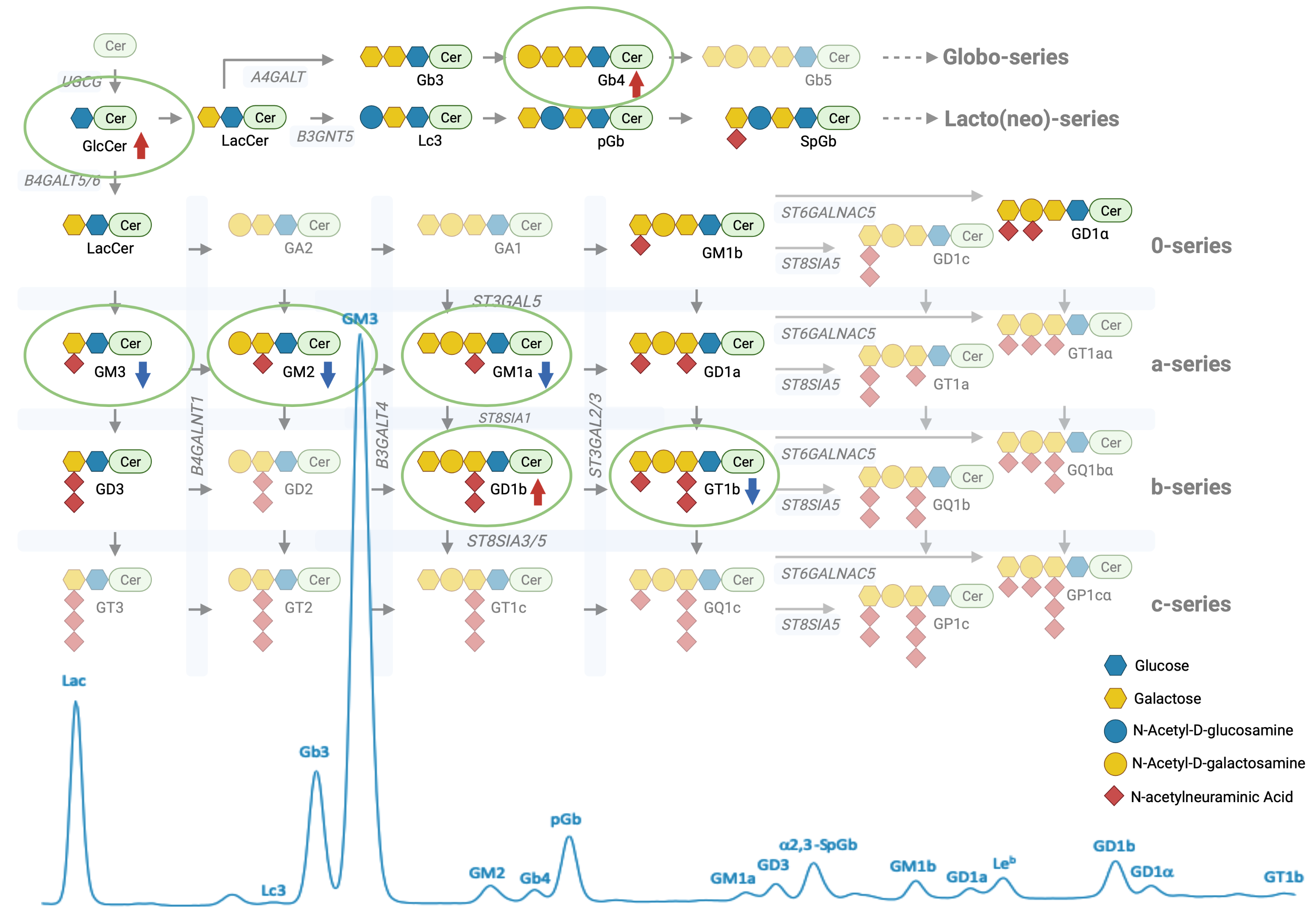

Supplement: Supplementary file 1 — Table S1. Glycosphingolipid levels. Table S2. Glycosphingolipid levels excluding GBA1 mutation carriers. Table S3. Comparison between GBA1 mutation carriers and noncarriers. Table S4. Glycosphingolipid levels in PD subjects stratified by gender and healthy control. Table S5. Glycosphingolipid levels in PD subjects with normal and high GlcCer. Table S6. Glycosphingolipid levels in PD subjects with normal and high GlcCer excluding GBA1 mutation carriers. Table S7. Correlations between GSLs and clinical features in PD subjects. Figure S1. Glycosphingolipid comparison between PD subjects and healthy controls. PD, Parkinson's disease; HC, healthy control. *P ≤ 0.05, **P < 0.01, ***P < 0.001, and ****P < 0.0001. Values inside the box are outliers. Figure S2. Glycosphingolipid biosynthetic pathway and representative high‐performance liquid chromatography profile and summary of main findings. HPLC trace of main plasma GSLs not included (GlcCer HPLC profile not shown). Green circles highlight abnormal glycosphingolipids in our analysis; red arrows indicate an increase in PD, whereas blue arrows indicate a decrease in PD. [file MDS-37-2129-s001.docx]
